# Long genes and genes with multiple splice variants are enriched in pathways linked to cancer and other multigenic diseases

**DOI:** 10.1186/s12864-016-2582-9

**Published:** 2016-03-12

**Authors:** Aleksandr B. Sahakyan, Shankar Balasubramanian

**Affiliations:** Department of Chemistry, University of Cambridge, Lensfield Road, Cambridge, CB2 1EW UK; Cancer Research UK Cambridge Institute, University of Cambridge, Li Ka Shing Centre, Robinson Way, Cambridge, CB2 0RE UK; School of Clinical Medicine, University of Cambridge, Cambridge, CB2 0SP UK

**Keywords:** Long genes, Splice variants, Cancer, Multigenic diseases, KEGG pathways, Mutations, Topoisomerases

## Abstract

**Background:**

The role of random mutations and genetic errors in defining the etiology of cancer and other multigenic diseases has recently received much attention. With the view that complex genes should be particularly vulnerable to such events, here we explore the link between the simple properties of the human genes, such as transcript length, number of splice variants, exon/intron composition, and their involvement in the pathways linked to cancer and other multigenic diseases.

**Results:**

We reveal a substantial enrichment of cancer pathways with long genes and genes that have multiple splice variants. Although the latter two factors are interdependent, we show that the overall gene length and splicing complexity increase in cancer pathways in a partially decoupled manner. Our systematic survey for the pathways enriched with top lengthy genes and with genes that have multiple splice variants reveal, along with cancer pathways, the pathways involved in various neuronal processes, cardiomyopathies and type II diabetes. We outline a correlation between the gene length and the number of somatic mutations.

**Conclusions:**

Our work is a step forward in the assessment of the role of simple gene characteristics in cancer and a wider range of multigenic diseases. We demonstrate a significant accumulation of long genes and genes with multiple splice variants in pathways of multigenic diseases that have already been associated with *de novo* mutations. Unlike the cancer pathways, we note that the pathways of neuronal processes, cardiomyopathies and type II diabetes contain genes long enough for topoisomerase-dependent gene expression to also be a potential contributing factor in the emergence of pathologies, should topoisomerases become impaired.

**Electronic supplementary material:**

The online version of this article (doi:10.1186/s12864-016-2582-9) contains supplementary material, which is available to authorized users.

## Background

Cancer is a complex family of multigenic diseases, where it is hard to single out a specific mechanism common to all its variants. Furthermore, a recent study [1] suggested that random replication errors play a major role in the emergence of cancer, with a correlation found between the number of cell divisions and the lifetime risk of cancer in different tissue types. Sixty-five percent of variation in the risk of cancer was shown to be explained by the number of cell divisions alone [1], with the heritable component explaining only up to 10 % of variation [2, 3]. There is also extensive evidence regarding the role of acquired *de novo* mutations in the autism spectrum disorder [4–6]. The involvement of long genes in autism was also noted [7], where the increased length was demonstrated to both multiply the probability of acquired mutations [8] and result in a decreased expression level of the long genes caused by impaired topoisomerases discovered to be crucial for the expression of the genes longer than 200 k nucleotides (nt) [7, 9].

The study of Tomasetti and Vogelstein [1] demonstrated the differential effect of random replication errors caused by a varying frequency of cell divisions in different tissues. In this work, we explore the possibility that even within a fixed number of cell divisions, there can still be differences in random mutation/genetic error burden of different genes and pathways, depending on the gene length and splicing complexity. We present analyses of all the genes in the human genome with a particular focus on the ones involved in cancer-linked pathways. We show that the gene length and splicing complexity are partially decoupled in defining their respective increase in cancer-linked pathways. Our work is a systematic study of the prior evidence of long genes involved in autism [7–9] and cancer [10], providing important evidence for the relevance of gene length in other multigenic diseases (cardiomyopathies, type II diabetes). In addition, we present the number of splice variants as another gene factor with significant overall increase in the pathways linked to multigenic diseases.

## Results and discussion

### Gene length and number of splice variants are increased in cancer pathways

**Fig. 1:**
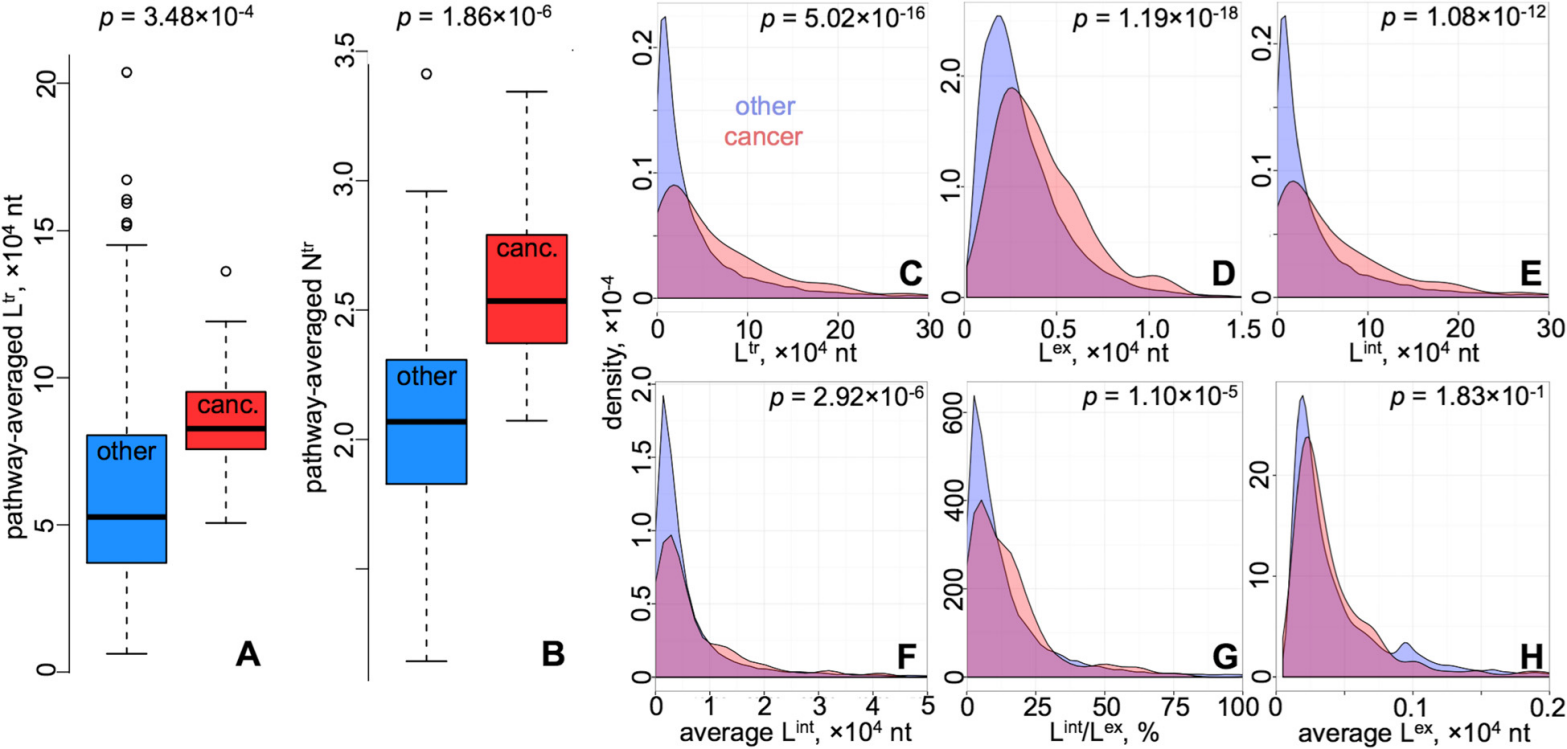
Enrichment of long transcripts and genes with greater number of transcript variants in cancer pathways. **a** Distribution of the pathway-averaged L^tr^ transcript length in cancer (red) and other (blue) pathways. **b** Distribution of the pathway-averaged N^tr^ number of transcripts in cancer (red) and other (blue) pathways. In the boxplots (**a**, **b**), each box is constructed via the median, first and third quartiles of the distribution. The whiskers show the range of values that are within the 1.5 times IQR (interquartile range). Individual points indicate the outliers. **c-h** Distributions of gene length and exon/intron composition descriptors in cancer (red) and other (blue) pathways. The plots are for the L^tr^ transcript (exons, UTR inclusive, and introns) length (**c**), L^ex^ summed exon length (**d**), L^int^ summed intron length (**e**), average L^int^ length of a single intron (**f**), L^int^/L^ex^ summed intron to summed exon length ratio (**g**) and average L^ex^ length of a single exon (**h**) for all the genes in other and cancer pathways. The *p*-values quantifying the significance of a positive shift in the distributions for the cancer pathways, as compared to others, are shown on top of each plot. Data from each of the **c-h** plots come from 380 cancer-linked and 18839 other genes

We explored the distribution of gene metrics in different pathways defined in the Kyoto encyclopaedia of genes and genomes (KEGG) [11], and found a marked increase in both the transcript length (Fig. 1a) and the number of transcripts (Fig. 1b), the latter reflecting the splice variants, for the genes in cancer pathways. In this context, the number of splice variants were analysed taking into account their partial dependence on gene length [12], as well as a number of studies where particular cancer variants were associated with impaired splicing [13–16].

The y-axes in Fig. 1a, b depict the values of the transcript length (L^tr^) and number of transcript variants (N^tr^) respectively, averaged for all the genes in each pathway across the cancer (15 pathways) and other (171 pathways) sets. For each gene, only the length of the longest transcript was considered. Genes in cancer pathways have on average a 86,250-nt-long transcript and 2.60 splice variants, as compared to 61,420 nt and 2.08 in other pathways (Table S1 in Additional file 1). The *p*-values demonstrating the significance of the positive shift in cancer pathways are shown in Fig. 1a, b. We used the Mann–Whitney nonparametric test, with the alternative hypothesis of the distribution average in cancer pathways being shifted towards greater values relative to the average of its comparison counterpart. The additional tests, comparing the numbers from randomly sampled equal numbers of pathways from cancer and other sets, confirmed the significance of the overall increase in L^tr^ and N^tr^ for the genes in cancer pathways (Figure S1 in Additional file 1).

In order to explore the factors that are behind the gene length increase in cancer pathways, we assessed the distributions of the L^tr^ transcript (coding sequence, UTRs and introns) length, L^ex^ summed exon (coding sequence and UTRs) length, L^int^ summed intron length, average L^int^ length (single intron), L^int^/L^ex^ total intron to exon length ratio, and average L^ex^ length (single exon) for all the genes in cancer and other pathways. Where multiple transcripts were present for a gene, data from its longest transcript was taken. The comparison of the distributions is shown in Fig. 1c-h, from where we can infer significant cancer-linked shifts in all the metrics for different gene elements (*p*-values: L^tr^, 5.02 × 10^−16^; L^ex^, 1.19 × 10^−18^; L^int^, 1.08 × 10^−12^; average L^int^, 2.92 × 10^−6^; L^int^/L^ex^, 1.10 × 10^−5^) except the average exon length (*p*-value: 0.183), which is rather similar in the genes involved in cancer vs. other pathways.

### Gene length and number of splice variants are increased in pathways of other multigenic diseases

**Fig. 2:**
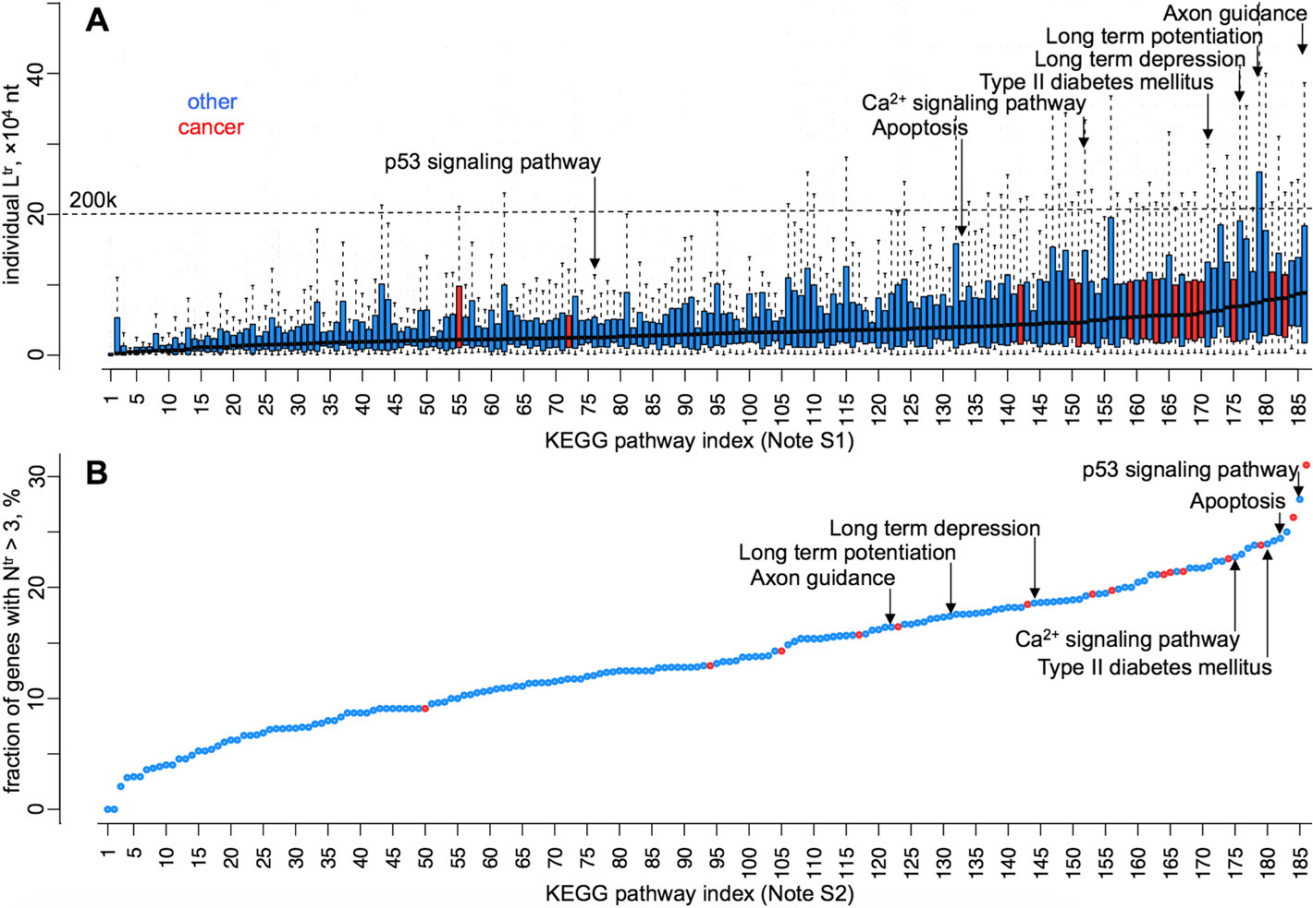
Genes in the pathways linked to multigenic diseases are, on average, longer and have more splice variants. **a** Distribution of L^tr^ in each of the 186 KEGG pathways, in the ascending order of the L^tr^ median values. The horizontal dashed line in (**a**) denotes the 200 k-nt threshold for a transcript length, known to be relevant in defining the topoisomerase-dependent transcription of the genes that are longer [7]. L^tr^ values are calculated for each unique gene, taking the length of the longest transcript, prior to splicing. **b** The fraction of genes (in an ascending order) with number of transcripts greater than 3 in each of the 186 KEGG pathways. The names of the KEGG pathways, ordered in the way corresponding to the indices in x-axes, can be found in Notes S1 and S2 in Additional file 1 for the plots (**a**) and (**b**). The cancer-linked pathways are highlighted in red, as opposed to blue for the rest. Some pathways from the rest, still related to cancer or other multigenic diseases are indicated with arrows

The distributions of L^tr^ transcript length (values corresponding to individual genes) involved in each KEGG pathway are shown in Fig. 2a. The cancer pathways are coloured in red. Some of the pathways that are markedly rich in long genes are associated with neuronal processes (see the labels in Fig. 2a), which could potentially be a contributing factor for the observed involvement of long genes in the etiology of autism spectrum disorder [7] (ASD). The link between 3 of the noted neurological KEGG pathways and ASD is further revealed via the enrichment analysis of the published 49 genes significantly associated with ASD (Table S2 in Additional file 1, similar analysis for gene ontology, GO, term enrichment is done in [7]), where we showed an enrichment of those genes in the long-term potentiation, long-term depression and Ca^2+^ signalling pathways. We used the DAVID gene annotation server [17, 18] for the enrichment analysis, with *Homo sapiens* genes set as the frequency background for normalisation.

We next investigated all KEGG pathways with regard to the fraction of genes that have greater than 3 splice variants (18.96 % from all the genes). The results are shown in Fig. 2b, where the pathways are arranged in ascending order of N^tr^ > 3 gene fraction. The cancer pathways are indeed accumulated in the rightmost side of the plot (red data points in Fig. 2b), containing more genes with multiple splice variants. Furthermore, among the other pathways rich in genes with multiple splice variants are p53 signaling and apoptosis (both associated with the etiology of cancer), as well as the same neuronal and type II diabetes mellitus pathways also enriched with long genes (compare the annotations in Fig. 2a and b).

### Cancer pathway enrichments with long genes and genes with multiple splice variants are partially decoupled

**Fig. 3:**
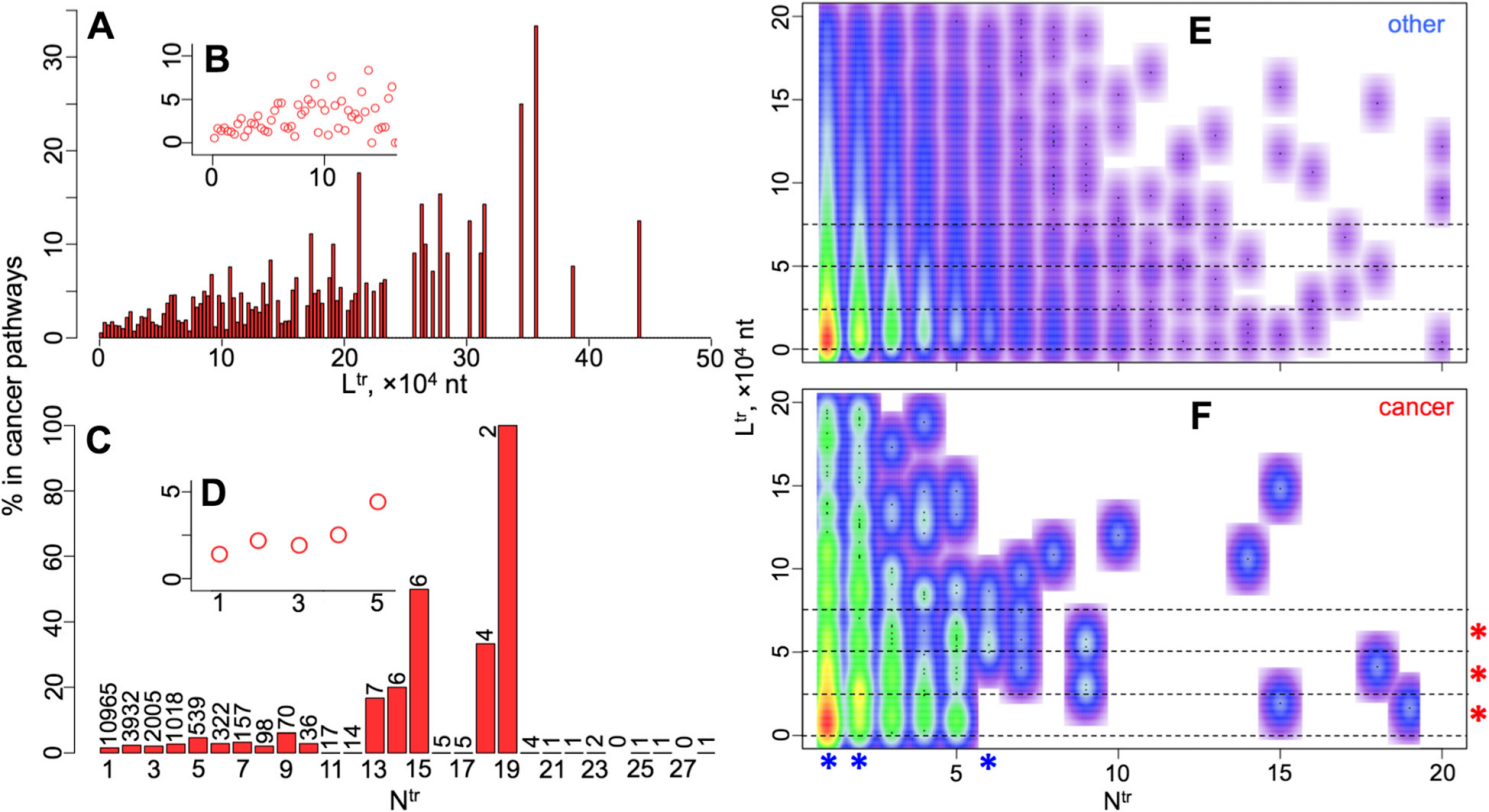
Gene length and number of splice variants are also decoupled in their linkage to the gene involvement in cancer pathways. **a** presents the L^tr^ (longest transcript length) of the genes via a 3000-nt window binning and shows the increase of the fraction of genes appearing in cancer pathways as L^tr^ increases. **b** The zoomed region below 1.6-mln-nt length for L^tr^. **c** shows the increase of the fraction of genes appearing in cancer pathways as the N^tr^ number of transcripts increase. The number of genes that have a given N^tr^ is shown on top of each bar. **d** The zoomed region below 6 for N^tr^, where more than 500 unique genes form each bar. **e**, **f** Density plots showing the density kernel estimates for the genes in other (**e**) and cancer (**f**) pathways respectively, calculated from the number of genes spread across varying L^tr^ (y-axes) and N^tr^ (x-axes). Please note, that the colour only denotes the density of the points, and, there is actually a positive correlation between L^tr^ and N^tr^ when taking the average values of L^tr^ [12] for each N^tr^ value. The red asterisks show the L^tr^ intervals where the positive N^tr^ shift in cancer vs. other pathways is significant. The blue asterisks show the N^tr^ values where the positive L^tr^ shift in cancer vs. other pathways is significant. For further details, see Figures S2 and S3 in Additional file 1

Above, we showed the increase of the overall gene length and the number of splice variants in cancer pathways. The same gradual shift can be noted while investigating the proportion of cancer-pathway-associated genes from all the genes found in different binned L^tr^ and N^tr^ intervals (Fig. 3a-d). However, since the gene length is also known to be positively linked to the number of splice variants [12], it is difficult to separate both effects from the data presented so far. We have, however, investigated the distributions of the genes involved in cancer and other pathways while stratifying our data and looking at either the L^tr^variation in different fixed N^tr^ categories (Figure S2 in Additional file 1) or the N^tr^ variation in relatively narrow fixed L^tr^ intervals (Figures S3 and S4 in Additional file 1).

In the two-dimensional representation of the gene count frequency with respect to L^tr^ and N^tr^ (y- and x-axes respectively in Fig. 3e, f), the stratified examination of the data is equivalent to comparing the outlined horizontal and vertical bands along both axes in Fig. 3e, f. Such comparison presented a significant positive shift, separately for both L^tr^ and N^tr^ variation, among the genes in the cancer vs. other pathways. The shift in gene length was significant when considering the genes with only 1 (*p*-value: 3.65 × 10^−7^), 2 (*p*-value: 1.23 × 10^−7^) and 6 (*p*-value: 3.64 × 10^−3^) splice variants (blue asterisks in Fig. 3f, Figure S2 in Additional file 1). Likewise, a significant positive shift in number of splice variants was noted for the genes stratified in 0 k-25 k (*p*-value: 1.62 × 10^−2^), 25 k-50 k (*p*-value: 7.24 × 10^−3^) and 50 k-75 k (*p*-value: 5.28 × 10^−4^) ranges of transcript length (red asterisks in Fig. 3f, Figure S3 in Additional file 1). The low significance of the other ranges for L^tr^ and N^tr^ can be attributed to fewer data coming from cancer pathways within those ranges.

### Pathways enriched in top genes by transcript length, summed exon length and number of splice variants

**Fig. 4:**
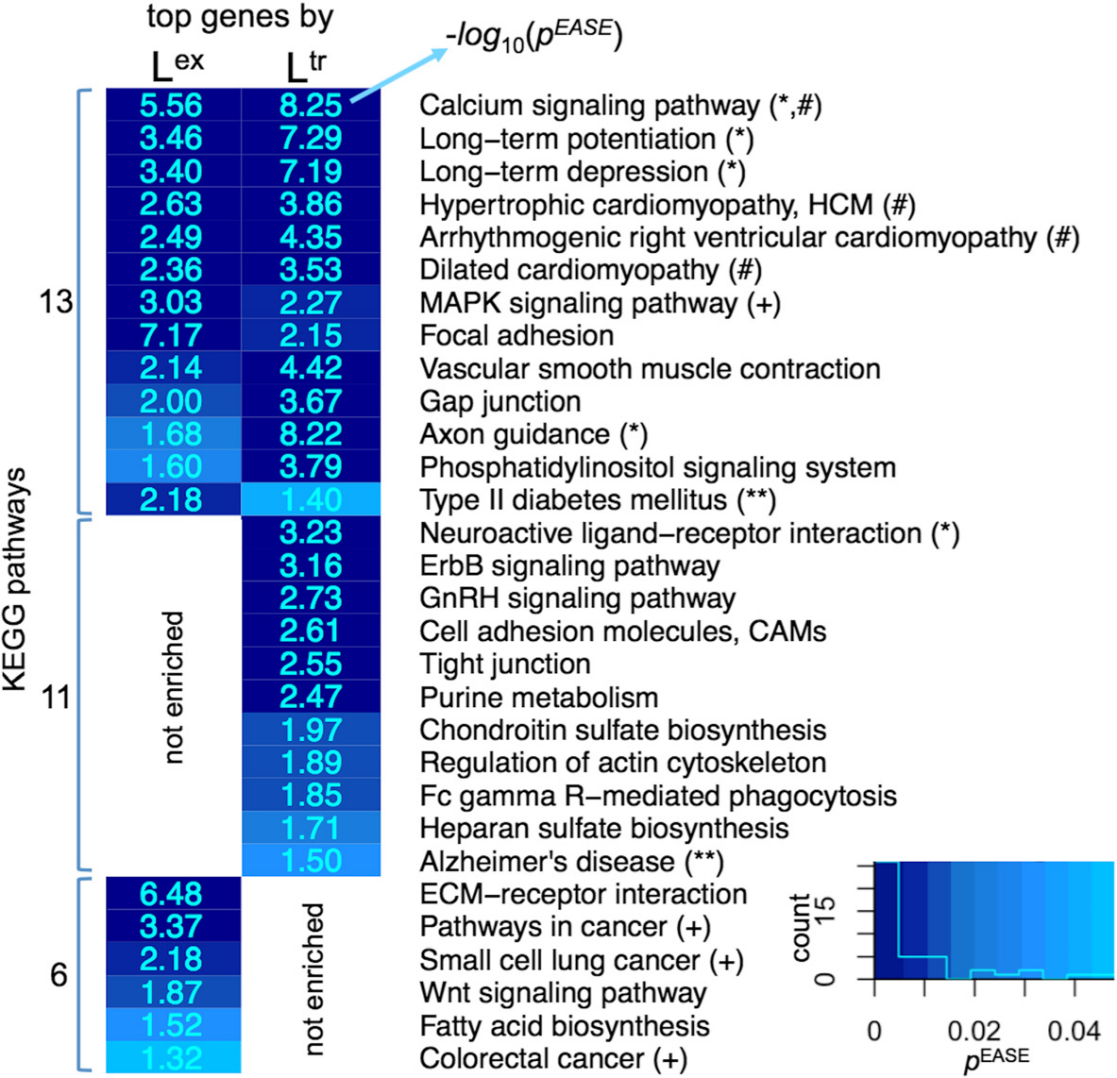
The KEGG pathway enrichment analysis for the top genes by L^ex^ summed exon and L^tr^ transcript lengths. Accounted for are the genes that have the L^ex^ or L^tr^ metrics (considered separately) greater than the median by twice the standard deviation. The significantly enriched KEGG pathways are revealed via DAVID gene functional annotation server, taking *Homo sapiens* as a correction background. The outcomes are presented as a heatmap, grouped by the presence/absence in both L^ex^ and L^tr^ categories, with individual rows ranked by the lowest significance score in {L^ex^, L^tr^} pair for each row. The *p* ^EASE^ significance scores for the enrichment are shown in a -*log*_*10*_ scale (−*log*_10_ *p* ^EASE^ > 1.301 means *p* ^EASE^ < 0.05), with the original distribution histogram and colour coding shown at the lower-right corner. The notations in the brackets mark the pathways linked to cancer (+), neurological (*), cardiological (#) and other (**) multigenic pathological conditions. The full list of genes that appear in each enriched pathway can be found in Additional file 3

We examined the top genes that have the longest summed exon (L^ex^) or the longest transcript (L^tr^) in our dataset (Additional file 2). For each category (L^ex^ and L^tr^), unique genes were selected out of all transcripts with the L^ex^ or L^tr^ (considered separately) being longer than the corresponding median value, by twice the standard deviation (roughly the top 2.3 % of data, Additional file 2). The pathway enrichment was then estimated via the DAVID gene annotation server [17, 18], with *Homo sapiens* genes set as the frequency background for normalisation. The resulting list of significantly enriched pathways is presented in Fig. 4, Tables S3 and S4 in Additional file 1.

As can be seen from the results, many of the revealed pathways are again linked to neuronal processes (marked with * in Fig. 4). The other two classes of KEGG pathways are the ones linked to cancer/cell differentiation (marked with + in Fig. 4), which are especially enriched in the genes with longest L^ex^, and, pathways associated with various cardiomyopathies (marked with # in Fig. 4), where there is a growing evidence [19–24] on the role of various *de novo* mutations in the family of diseases. Furthermore, type II diabetes mellitus, another multigenic disease [25, 26] the linked KEGG pathway of which is enriched with long genes (marked with ** in Fig. 4), is revealed again. Interestingly, the neurological pathways appear to be more enriched with the top genes by longest L^tr^ transcripts, as compared to L^ex^ summed exon. This may indicate the presence of general selection in neurological pathways favouring longer transcripts (irrespective of the summed exon length), potentially, to accommodate additional control mechanisms for gene regulation at the DNA level, achievable due to the noted specificities (for instance, topoisomerase involvement) in the transcription of the long genes [7, 9].

The KEGG pathway enrichment analysis for the genes that have more than 3 transcript variants is summarised in Table 1, showing many cancer-linked pathways along with the Ca^2+^ signaling pathway. The latter may have roles in both ASD (Table S2 in Additional file 1) and cardiomyopathies.

**Table 1:** The KEGG pathway enrichment analysis for the genes with number of transcripts greater than 3

Genes with N^tr^ > 3		
KEGG pathway	Number of genes	*p* ^EASE^ score
Calcium signaling pathway (^b,c^)	40	8.43 10^−4^
Pathways in cancer (^a^)	64	1.54 10^−3^
p53 signaling pathway (^a^)	19	2.93 10^−3^
MAPK signaling pathway (^a^)	51	7.68 10^−3^
Apoptosis (^a^)	21	9.85 10^−3^
Acute myeloid leukemia (^a^)	15	1.88 10^−2^
ErbB signaling pathway	20	2.00 10^−2^
NOD-like receptor signaling pathway	15	3.25 10^−2^
Thyroid cancer (^a^)	9	3.29 10^−2^
Progesterone-mediated oocyte maturation	19	3.44 10^−2^
Hematopoietic cell lineage	19	3.44 10^−2^
Neurotrophin signaling pathway	25	3.87 10^−2^
Wnt signaling pathway	29	4.45 10^−2^
Prostate cancer (^a^)	19	4.65 10^−2^
Adherens junction	17	4.71 10^−2^

The significantly enriched KEGG pathways are revealed via DAVID gene functional annotation server, taking *Homo sapiens* as a correction background. The *p* ^EASE^ significance scores for the enrichment are shown along with the number of hit genes. The notations in the brackets mark the pathways linked to cancer (^a^), neurological (^b^) and cardiological (^c^) conditions. The full list of genes that appear in each enriched pathway can be found in Additional file 3

Since the genes in KEGG pathways are manually curated to have high consistency and close link to the underlying biochemical network, we have used the KEGG pathway enrichment outcomes throughout the discussion. However, we also performed similar analyses using gene ontology (GO) terms [27], revealing many terms that are related to the found KEGG pathways (Figure S5 in Additional file 1). The full set of results from both KEGG and GO enrichment analyses can be found in Additional file 3.

### Number of somatic mutations found in different genes is correlated with gene length

**Fig. 5:**
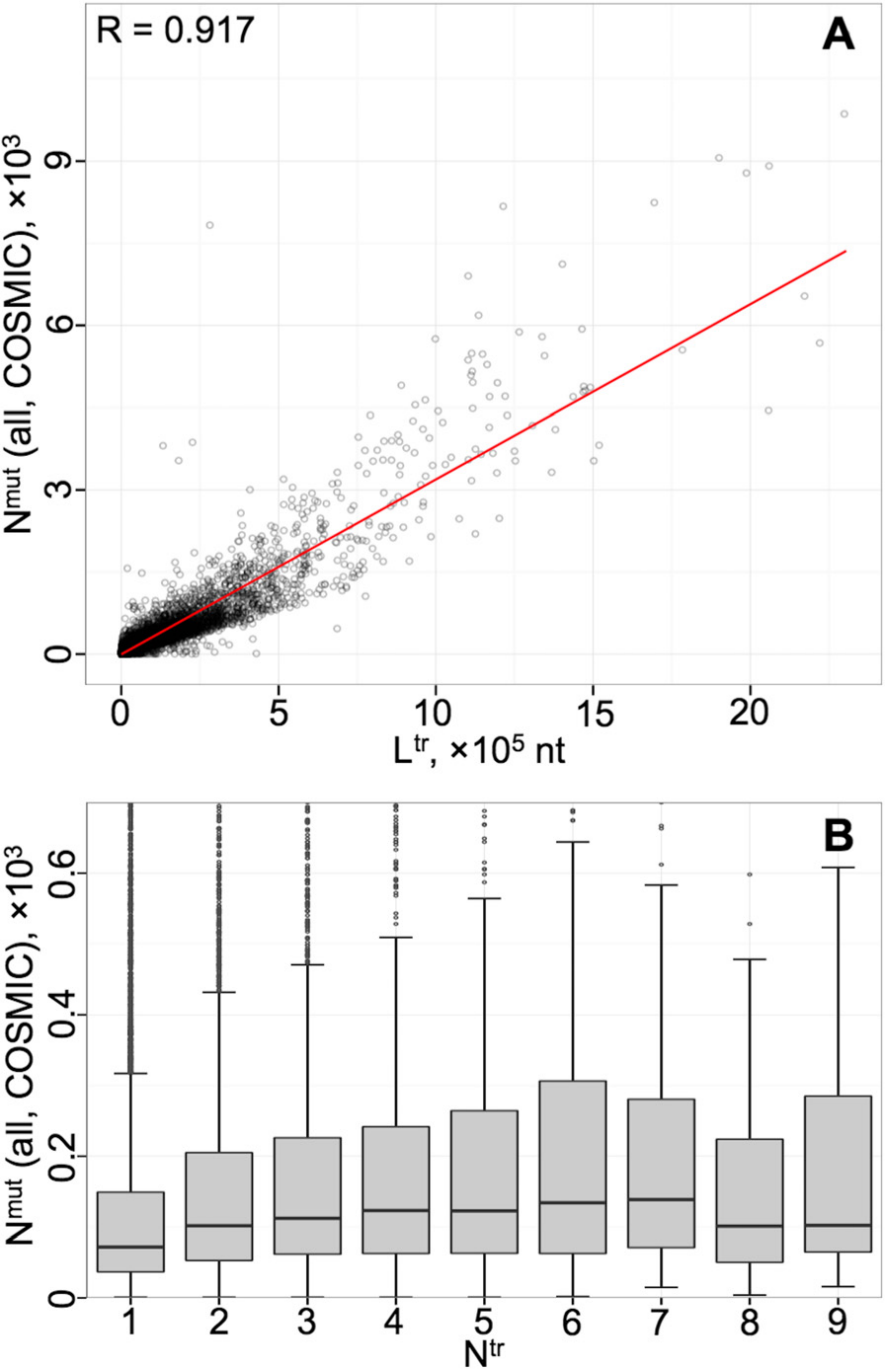
The relations among the overall number of recorded cancer-linked somatic mutations, transcript length and number of splice variants. **a** Linear correlation between the overall number of cancer-linked mutations in different genes and their transcript length. The cancer-linked somatic mutations (as deposited in the COSMIC database) are counted from both the coding and non-coding regions, for the longest transcript of each gene, and plotted against the L^tr^ length of that transcript. The correlation coefficient (top-left corner) and the linear model fit (red line) are shown. **b** Relation between the overall number of cancer-linked mutations and the number of N^tr^ transcript variants in different genes. The individual boxplots (for each N^tr^ category) describe the distribution of the overall number of mutations found in the longest transcript of each gene, selected from the genes with fixed N^tr^ number of available transcripts

One of the ways a long gene can have more pronounced involvement in multigenic diseases could be through the increased propensity for mutation. The longer the gene, the higher the probability that within a certain number of replication events (cell divisions) the gene may acquire a mutation, as also reflected in the accumulated and fixated germline mutations [28]. To directly demonstrate this non-specific link between the number of somatic mutations found in different genes and the gene length, we explored the genome-wide set of cancer-linked somatic mutations, deposited in the COSMIC database [29]. Please note, it is hard to differentiate which mutations are causing cancer and which are the consequences of cancer in such datasets, hence this analysis is only for demonstrating the link between the number of mutations and gene length, rather than for drawing quantitative conclusions. It complements the above KEGG pathway exploration, where the cancer pathways are manually curated to contain whole gene networks with members consistently linked to the pathogeneses of different types of cancer. The outcome for the number of somatic mutations is presented in Fig. 5a while jointly considering both coding and non-coding mutations against the transcript (containing all exons, UTR inclusive, and introns) length.

A strong correlation is noted (Pearson’s *R* = 0.917), with, on average, 3.2 mutations per 1000-nt-long transcript (mRNA coding genes) recorded in the COSMIC database. Similarly, the number of mutations that occur only within coding sequences (CDS) correlate with the CDS lengths (*R* = 0.871), as presented in Figure S6 in Additional file 1. For the latter dependence, there are, on average, 33.17 mutations recorded per 1000-nt-long CDS, which might be the reflection of both a greater rate of spontaneous mutations in exons [30] and the more comprehensive exploration and greater amount of recorded data for exons in the COSMIC database owing to the application of predominantly exome-targeted sequencing techniques. To this end, such comparisons between mutation numbers inferred while comparing different datasets or different parts of genes from the same dataset are not conclusive and should be carried out with caution. Figures S7A, B in Additional file 1 present the versions of Fig. 5a and Figure S6 zoomed on the densely populated regions. We also show the relation between the numbers of somatic mutations and of splice variants (for the N^tr^ groups with significant number of data, as seen in Fig. 3c), which reflects a certain degree of proportional dependence (Fig. 5b).

## Conclusions

Our results highlight that the pathways linked to cancer and other multigenic diseases are enriched with long genes and genes that have increased number of splice variants (Figs. 1, 2 and 3a-d). The observation of the latter enrichment generalises and reinforces the prior proposals of the splicing process as one of the cancer-causing factors, if impaired [13–16]. Taking into account the presence of a directly proportional dependence between the gene length and the number of splice variants [12], we have taken additional steps to demonstrate (Fig. 3e, f, Figures S2 and S3 in Additional file 1) that the significance of both factors in defining the gene presence in cancer pathways are in part decoupled from each other.

We showed that the overall increase in gene length in cancer pathways is accompanied by the increase in both summed exon (L^ex^) and intron (L^int^) lengths. The L^int^/L^ex^ratio is elevated as well (Fig. 1c-h), indicating a non-proportional increase in intron over exon sizes, most probably associated with the retrotransposonal infiltration of genes [12], where the transposable elements are less fatal (hence pass on to generations) while inserted within introns.

We presented a systematic survey of all the KEGG pathways for long genes and genes with multiple splice variants (Figs. 2 and 3, Table 1, Tables S2, S3 and S4 in Additional file 1). In addition to cancer pathways, the results showed a significant presence of long genes and genes with multiple splice variants in pathways linked to neuronal processes that may have a role in ASD, cardiomyopathies, and type II diabetes - all complex multigenic diseases with myriads of evidence on their link with the acquisition of different *de novo* mutations [4–6, 19–26].

We analysed the genome-wide data on the reported 8.4 million cancer-linked somatic mutations, demonstrating a logically expected link between the gene length and the number of recorded somatic mutations (Fig. 5, Figures S6 and S7 in Additional file 1). This points out that long genes might simply have higher probabilities to incur a mutation. There are, however, many other ways for the long genes to become associated with multigenic diseases. Long genes may provide more options for interactions with other gene products (such as transcription factors, RNA-binding proteins, non-coding RNAs and other regulators targeting particular sequences), hence increasing the number of factors that can potentially affect their expression and integrity [31]. It has recently been discovered that topoisomerases play a role in the expression of genes longer than 200 k nt [7], perhaps owing to the necessity to remove supercoiled structures in long DNA segments to be transcribed. This introduces yet another mechanism by which the homeostasis of long genes can become vulnerable due to possible impairments in topoisomerases. We show that, although this mechanism may be relevant for the neurological pathways, cardiomyopathies and type II diabetes (Fig. 2a, Note S1 in Additional file 1), all of which contain genes longer than 200 k, the mechanism is probably not definitive for cancer pathways, as most of the genes there are below the 200 k-nt threshold for the length (Fig. 2a).

Overall, the outcomes of this study extend our understanding of how simple characteristics of genes can associate with cancer and a wider range of multigenic diseases. We anticipate the combined usage of the gene length and the number of splice variants to become an important component in the algorithms for identifying novel genes with significant risks of association with multigenic diseases, where we can also take advantage of the availability of intrinsic, context-dependent probabilities for nucleotide substitutions at all the base positions in each gene [32].

## Methods

All the analyses were done using the R programming language and data analysis environment [33]. The underlying scripts are available from the authors upon request.

### Calculation of the gene size and exon/intron metrics

The full gene list and position information were taken from the annotation tables in the UCSC genome browser corresponding to the human reference genome, sequence version GRCh37. Only the nuclear genome was considered, with the analysis done for the 37,559 mRNA-coding transcripts. For each transcript, its length (l^tr^), number of exons (n^ex^), number of introns (n^int^), total exon length (l^ex^), total intron length (l^int^), average exon length (l^ex^/n^ex^), average intron length (l^int^/n^int^) and the intron to exon length ratio (l^int^/l^ex^) were calculated. The complete data can be accessed in Additional file 2. For the further analyses, where multiple transcripts were present for the same gene, the single longest transcript or the first transcript from top equal-length ones was picked, resulting in 19,219 transcripts (with the length denoted as L^tr^) corresponding to unique genes. The number of all available transcripts for each gene was taken as N^tr^ descriptor of splicing complexity.

### KEGG pathway assignment of the genes

To assign the genes to one of the functional pathways, the gene sets derived from the KEGG [11] pathway database were taken from the Molecular Signature Database [34] (http://www.broadinstitute.org/gsea/msigdb accessed in September 2014). The data contained 186 sets, each corresponding to distinct KEGG pathways. The full names of the KEGG pathways can be found in the Notes S1 and S2 in Additional file 1, brought in the order corresponding to the pathway indices in Fig. 2a and b respectively.

### Exploratory data analyses and statistics

Further exploratory boxplots and histograms were created with R base and ggplot2 [35] libraries. The cancer and other pathway distributions in Fig. 1c-h, Figures S2, S3 and S4 in Additional file 1 are comparably visualised by taking the density (y-axes) calculated via a Gaussian kernel, instead of direct counts. This was done using the default settings of the geom_density function in the ggplot2 library. The 2-dimensional histograms in Fig. 2e, f were created by calculating the 2D binned kernel density estimates (bkde2D function of the KernSmooth [36] library), binning both the x- and y-axes by 600 equally spaced points. The density plots and associated density estimations are useful for the unbiased comparison of two distributions where the data points do not necessarily span the same range of values (same span of x-axis) and/or are unequally spaced. Please note, that such representations have no effect on our *p*-value calculations for the significance in differences between distributions, since for the latter we used the full set of actual data values (x-axis values) for each distribution. To assess the significance of the variation in transcript length and number of transcripts while comparing distributions in cancer and other pathways (Fig. 1a-h, Figures S2 and S3 in Additional file 1), *p*-values were calculated using the Mann–Whitney nonparametric test, which has a greater efficiency than the standard *t*-test for the distributions that deviate from the normal ones, and has efficiency close to the *t*-test for normal distributions. The null hypothesis in the test was that the average value from the distribution in cancer pathways is not greater than the value in other pathways.

### KEGG and GO enrichment analyses of the ASD genes, top genes by length and genes with multiple splice variants

The 49 genes significantly associated with ASD (FDR < 0.05) were taken from [7]. For the gene length, we separately considered the top genes by their summed exon (L^ex^) and transcript (L^tr^) lengths. For the top genes by L^ex^ and L^tr^, we took the genes with those metrics being greater than the corresponding median value plus 2 times the standard deviation. This resulted in 986 and 802 genes for the top L^ex^ and L^tr^ respectively. For the genes with multiple splice variants, we took the genes that have more than 3 transcripts (N^tr^ > 3), resulting in 2317 genes (18.96 % of data). The described gene sets were then used to detect pathway enrichment via the DAVID [17, 18] server for gene functional annotation (Fig. 4, Table 1, Tables S2, S3 and S4, and Figure S5 in Additional file 1). We considered the significantly enriched KEGG [11] pathways and GO terms [27], normalising the results against the background frequencies of all the genes in *Homo sapiens*. A pathway was considered significantly enriched, if possessing a *p*^EASE^-score (modified exact Fischer *p*-value) of less than 0.05, as recommended at DAVID [17, 18]. The full set of results from both KEGG and GO enrichment analyses is deposited in Additional file 3.

### Analysis of the cancer-linked mutations observed for different genes

To directly explore the link between the number of somatic mutations and the gene length, we used all the observed cancer-linked mutations of different types through the COSMIC database [29] (v72, accessed in May 2015) of somatic mutations in cancer. Datasets for the mutations at both coding (CodingMutantExport.tsv) and non-coding (CosmicNCV.tsv) regions were merged and trimmed to remove the repeated mutational event observations for the same chromosome and position. This resulted in 8,399,914 events corresponding to unique genomic positions annotated for the RefSeq version GRCh38, of which 7,238,632 (86.2 %) originate from the non-coding variation dataset. The positions were then mapped onto the transcripts, with the border coordinates retrieved for the matching GRCh38 version of the human genome from the UCSC genome browser. Only the genes from the nuclear genome were considered. Whenever the query mutation site was engulfed by more than one transcript, only a single first appearing transcript was assigned. Next, the total number of mutation events was calculated for each transcript. The results were then superimposed to obtain a single value for the number of cancer-linked mutations per gene, by taking the value from only the longest transcript for each unique gene name. This resulted in a set of 18,204 genes with associated number of mutation events reported in the COSMIC database. A linear model fitting for the number of mutations versus L^tr^ dependence in Fig. 5 resulted in −3.4 intercept and 0.00320 slope, showing that a 1000-nt-long transcript has on average 3.20 mutations reported in COSMIC. We also studied the relationship between the mutations occurring only at coding regions (13.8 % of data) and the coding sequence (CDS) length as reported in the CodingMutantExport.tsv file at COSMIC (Figure S6 in Additional file 1). For this dependence, COSMIC database contained, on average, 33.17 mutations per 1000-nt-long CDS, most probably as a result of more detailed exploration, hence more reported mutations, for coding sequences owing to the exome-only sequencing studies.

## Additional files

Additional file 1: Supplementary Notes S1 and S2, Tables S1-S4 and Figures S1-S7. The file is in the PDF format. The content is individually referred in the main text. (PDF 1637 kb) Additional file 2: The calculated lengths (nt) of all the mRNA-coding nuclear transcripts (GRCh37) along with the number of exons, introns and their length fractions. The file is in the comma-separated CSV format. (CSV 2851 kb) Additional file 3: All the KEGG pathway and GO term analyses data used in this work. KEGG pathways and GO terms significantly enriched in the top genes by the transcript length, top genes by summed exon length, genes with greater than 3 transcript variants, and KEGG pathways significantly enriched in ASD-associated genes are presented. The data include the full list of the hit gene names. The file is in the Excel XLSX format. (XLSX 99 kb)

## Abbreviations

2D: 2-dimensional
ASD: Autism spectrum disorder
CDS: Coding sequence
COSMIC: Catalogue of somatic mutations in cancer
GO: Gene ontology
KEGG: Kyoto encyclopaedia of genes and genomes
nt: Nucleotide
UTR: Untranslated region
