# Supplementary material for: Long genes and genes with multiple splice variants are enriched in pathways linked to cancer and other multigenic diseases
Source: BMC Genomics. 2016 Mar 12;17:225. doi: 10.1186/s12864-016-2582-9 (PMC4788956; doi:10.1186/s12864-016-2582-9)
Supplement: Additional file 1: — Supplementary Notes S1 and S2, Tables S1-S4 and Figures S1-S7. The file is in the PDF format. The content is individually referred in the main text. (PDF 1637 kb) [file 12864_2016_2582_MOESM1_ESM.pdf]

Long genes and genes with multiple splice variants are enriched in pathways linked to cancer and other multigenic diseases

Aleksandr B. Sahakyan[1,2] and Shankar Balasubramanian[1,2,3,*]

[1] Department of Chemistry, University of Cambridge, Lensfield Road, Cambridge CB2 1EW, UK.
[2] Cancer Research UK Cambridge Institute, University of Cambridge, Li Ka Shing Centre, Robinson Way, Cambridge CB2 0RE, UK.
[3] School of Clinical Medicine, University of Cambridge, Cambridge CB2 0SP, UK.
* Correspondence: sb10031@cam.ac.uk

Additional file 1: Supplementary Notes, Tables and Figures

Contents

Note S1 ---- 2
Note S2 ---- 5
Table S1 ---- 8
Table S2 ---- 9
Table S3 ---- 10
Table S4 ---- 11
Figure S1 ---- 12
Figure S2 ---- 13
Figure S3 ---- 14
Figure S4 ---- 15
Figure S5 ---- 16
Figure S6 ---- 17
Figure S7 ---- 18

Note S1 The list of KEGG pathway names, in the same order as in Figure 2A

1 - OLFACTORY_TRANSDUCTION
2 - TASTE_TRANSDUCTION
3 - SYSTEMIC_LUPUS_ERYTHEMATOSUS
4 - RIBOSOME
5 - AUTOIMMUNE_THYROID_DISEASE
6 - ASTHMA
7 - ALLOGRAFT_REJECTION
8 - MATURITY_ONSET_DIABETES_OF_THE_YOUNG
9 - GRAFT_VERSUS_HOST_DISEASE
10 - ANTIGEN_PROCESSING_AND_PRESENTATION
11 - CYTOSOLIC_DNA_SENSING_PATHWAY
12 - TYPE_I_DIABETES_MELLITUS
13 - REGULATION_OF_AUTOPHAGY
14 - OXIDATIVE_PHOSPHORYLATION
15 - PARKINSONS_DISEASE
16 - FOLATE_BIOSYNTHESIS
17 - INTESTINAL_IMMUNE_NETWORK_FOR_IGA_PRODUCTION
18 - CYTOKINE_CYTOKINE_RECEPTOR_INTERACTION
19 - BASE_EXCISION_REPAIR
20 - GLUTATHIONE_METABOLISM
21 - PRIMARY_IMMUNODEFICIENCY
22 - LEISHMANIA_INFECTION
23 - RIG_I_LIKE_RECEPTOR_SIGNALING_PATHWAY
24 - PROTEASOME
25 - ARACHIDONIC_ACID_METABOLISM
26 - ALZHEIMERS_DISEASE
27 - HUNTINGTONS_DISEASE
28 - SPLICEOSOME
29 - RNA_POLYMERASE
30 - GLYCOSYLPHOSPHATIDYLINOSITOL_GPI_ANCHOR_BIOSYNTHESIS
31 - TOLL_LIKE_RECEPTOR_SIGNALING_PATHWAY
32 - PATHOGENIC_ESCHERICHIA_COLI_INFECTION
33 - NATURAL_KILLER_CELL_MEDIATED_CYTOTOXICITY
34 - METABOLISM_OF_XENOBIOTICS_BY_CYTOCHROME_P450
35 - OTHER_GLYCAN_DEGRADATION
36 - HEMATOPOIETIC_CELL_LINEAGE
37 - VALINE_LEUCINE_AND_ISOLEUCINE_BIOSYNTHESIS
38 - PROXIMAL_TUBULE_BICARBONATE_RECLAMATION
39 - CARDIAC_MUSCLE_CONTRACTION
40 - SULFUR_METABOLISM
41 - GLYCOLYSIS_GLUCONEOGENESIS
42 - GLYCOSPHINGOLIPID_BIOSYNTHESIS_LACTO_AND_NEOLACTO_SERIES
43 - PRION_DISEASES
44 - NEUROACTIVE_LIGAND_RECEPTOR_INTERACTION
45 - ALPHA_LINOLENIC_ACID_METABOLISM
46 - LINOLEIC_ACID_METABOLISM
47 - TYROSINE_METABOLISM
48 - DRUG_METABOLISM_CYTOCHROME_P450
49 - PPAR_SIGNALING_PATHWAY
50 - RNA_DEGRADATION
51 - TERPENOID_BACKBONE_BIOSYNTHESIS
52 - GLYCINE_SERINE_AND_THREONINE_METABOLISM
53 - NOD_LIKE_RECEPTOR_SIGNALING_PATHWAY
54 - GLYCOSAMINOGLYCAN_BIOSYNTHESIS_KERATAN_SULFATE
55 - BLADDER_CANCER
56 - PEROXISOME
57 - ETHER_LIPID_METABOLISM
58 - RETINOL_METABOLISM
59 - STEROID_BIOSYNTHESIS
60 - JAK_STAT_SIGNALING_PATHWAY
61 - PYRIMIDINE_METABOLISM
62 - CHEMOKINE_SIGNALING_PATHWAY
63 - GLYOXYLATE_AND_DICARBOXYLATE_METABOLISM
64 - DNA_REPLICATION
65 - PORPHYRIN_AND_CHLOROPHYLL_METABOLISM
66 - NITROGEN_METABOLISM
67 - PYRUVATE_METABOLISM
68 - ARGININE_AND_PROLINE_METABOLISM
69 - PHENYLALANINE_METABOLISM
70 - COMPLEMENT_AND_COAGULATION_CASCADES
71 - VIRAL_MYOCARDITIS
72 - BASAL_CELL_CARCINOMA
73 - CELL_ADHESION_MOLECULES_CAMS

74 - FATTY_ACID_METABOLISM
75 - CITRATE_CYCLE_TCA_CYCLE
76 - P53_SIGNALING_PATHWAY
77 - TAURINE_AND_HYPOTAURINE_METABOLISM
78 - LYSOSOME
79 - HISTIDINE_METABOLISM
80 - SNARE_INTERACTIONS_IN_VESICULAR_TRANSPORT
81 - N_GLYCAN_BIOSYNTHESIS
82 - SELENOAMINO_ACID_METABOLISM
83 - FRUCTOSE_AND_MANNOSE_METABOLISM
84 - AMINO_SUGAR_AND_NUCLEOTIDE_SUGAR_METABOLISM
85 - CYSTEINE_AND_METHIONINE_METABOLISM
86 - RENIN_ANGIOTENSIN_SYSTEM
87 - STEROID_HORMONE_BIOSYNTHESIS
88 - PRIMARY_BILE_ACID_BIOSYNTHESIS
89 - PENTOSE_AND_GLUCURONATE_INTERCONVERSIONS
90 - VASOPRESSIN_REGULATED_WATER_REABSORPTION
91 - AMYOTROPHIC_LATERAL_SCLEROSIS_ALS
92 - PROTEIN_EXPORT
93 - DRUG_METABOLISM_OTHER_ENZYMES
94 - GLYCOSPHINGOLIPID_BIOSYNTHESIS_GLOBO_SERIES
95 - HEDGEHOG_SIGNALING_PATHWAY
96 - CELL_CYCLE
97 - ASCORBATE_AND_ALDARATE_METABOLISM
98 - NUCLEOTIDE_EXCISION_REPAIR
99 - PENTOSE_PHOSPHATE_PATHWAY
100 - PANTOTHENATE_AND_COA_BIOSYNTHESIS
101 - BUTANOATE_METABOLISM
102 - RIBOFLAVIN_METABOLISM
103 - BASAL_TRANSCRIPTION_FACTORS
104 - ALANINE_ASPARTATE_AND_GLUTAMATE_METABOLISM
105 - LIMONENE_AND_PINENE_DEGRADATION
106 - GLYCEROLIPID_METABOLISM
107 - VIBRIO_CHOLERAE_INFECTION
108 - VEGF_SIGNALING_PATHWAY
109 - GNRH_SIGNALING_PATHWAY
110 - MELANOGENESIS
111 - AMINOACYL_TRNA_BIOSYNTHESIS
112 - NOTCH_SIGNALING_PATHWAY
113 - PURINE_METABOLISM
114 - HOMOLOGOUS_RECOMBINATION
115 - HYPERTROPHIC_CARDIOMYOPATHY_HCM
116 - GLYCEROPHOSPHOLIPID_METABOLISM
117 - ONE_CARBON_POOL_BY_FOLATE
118 - MISMATCH_REPAIR
119 - GLYCOSAMINOGLYCAN_DEGRADATION
120 - SPHINGOLIPID_METABOLISM
121 - TRYPTOPHAN_METABOLISM
122 - TGF_BETA_SIGNALING_PATHWAY
123 - OOCYTE_MEIOSIS
124 - MAPK_SIGNALING_PATHWAY
125 - VALINE_LEUCINE_AND_ISOLEUCINE_DEGRADATION
126 - GALACTOSE_METABOLISM
127 - LEUKOCYTE_TRANSENDOTHELIAL_MIGRATION
128 - ADIPOCYTOKINE_SIGNALING_PATHWAY
129 - PROPANOATE_METABOLISM
130 - ALDOSTERONE_REGULATED_SODIUM_REABSORPTION
131 - LYSINE_DEGRADATION
132 - GLYCOSAMINOGLYCAN_BIOSYNTHESIS_CHONDROITIN_SULFATE
133 - APOPTOSIS
134 - ENDOCYTOSIS
135 - BIOSYNTHESIS_OF_UNSATURATED_FATTY_ACIDS
136 - EPITHELIAL_CELL_SIGNALING_IN_HELICOBACTER_PYLORI_INFECTION
137 - FC_EPSILON_RI_SIGNALING_PATHWAY
138 - STARCH_AND_SUCROSE_METABOLISM
139 - INSULIN_SIGNALING_PATHWAY
140 - WNT_SIGNALING_PATHWAY
141 - NICOTINATE_AND_NICOTINAMIDE_METABOLISM
142 - PATHWAYS_IN_CANCER
143 - UBIQUITIN_MEDIATED_PROTEOLYSIS
144 - BETA_ALANINE_METABOLISM
145 - CIRCADIAN_RHYTHM_MAMMAL
146 - PROGESTERONE_MEDIATED_OOCYTE_MATURATION
147 - VASCULAR_SMOOTH_MUSCLE_CONTRACTION
148 - TIGHT_JUNCTION
149 - GAP_JUNCTION

150 - THYROID_CANCER
151 - MELANOMA
152 - CALCIUM_SIGNALING_PATHWAY
153 - NEUROTROPHIN_SIGNALING_PATHWAY
154 - NON_HOMOLOGOUS_END_JOINING
155 - GLYCOSPHINGOLIPID_BIOSYNTHESIS_GANGLIO_SERIES
156 - GLYCOSAMINOGLYCAN_BIOSYNTHESIS_HEPARAN_SULFATE
157 - T_CELL_RECEPTOR_SIGNALING_PATHWAY
158 - MTOR_SIGNALING_PATHWAY
159 - COLORECTAL_CANCER
160 - REGULATION_OF_ACTIN_CYTOSKELETON
161 - CHRONIC_MYELOID_LEUKEMIA
162 - INOSITOL_PHOSPHATE_METABOLISM
163 - PROSTATE_CANCER
164 - FC_GAMMA_R_MEDIATED_PHAGOCYTOSIS
165 - DILATED_CARDIOMYOPATHY
166 - PANCREATIC_CANCER
167 - B_CELL_RECEPTOR_SIGNALING_PATHWAY
168 - ACUTE_MYELOID_LEUKEMIA
169 - SMALL_CELL_LUNG_CANCER
170 - RENAL_CELL_CARCINOMA
171 - TYPE_II_DIABETES_MELLITUS
172 - ECM_RECEPTOR_INTERACTION
173 - O_GLYCAN_BIOSYNTHESIS
174 - FOCAL_ADHESION
175 - GLIOMA
176 - LONG_TERM_DEPRESSION
177 - PHOSPHATIDYLINOSITOL_SIGNALING_SYSTEM
178 - ABC_TRANSPORTERS
179 - LONG_TERM_POTENTIATION
180 - ARRHYTHMOGENIC_RIGHT_VENTRICULAR_CARDIOMYOPATHY_ARVC
181 - ENDOMETRIAL_CANCER
182 - ERBB_SIGNALING_PATHWAY
183 - NON_SMALL_CELL_LUNG_CANCER
184 - DORSO_VENTRAL_AXIS_FORMATION
185 - ADHERENS_JUNCTION
186 - AXON_GUIDANCE

Note S2 The list of KEGG pathway names, in the same order as in Figure 2B

1 - LIMONENE_AND_PINENE_DEGRADATION
2 - ASTHMA
3 - OLFACTORY_TRANSDUCTION
4 - ALLOGRAFT_REJECTION
5 - BUTANOATE_METABOLISM
6 - REGULATION_OF_AUTOPHAGY
7 - HISTIDINE_METABOLISM
8 - O_GLYCAN_BIOSYNTHESIS
9 - GLYCOSAMINOGLYCAN_BIOSYNTHESIS_HEPARAN_SULFATE
10 - ASCORBATE_AND_ALDARATE_METABOLISM
11 - AUTOIMMUNE_THYROID_DISEASE
12 - BETA_ALANINE_METABOLISM
13 - GLYCOSAMINOGLYCAN_BIOSYNTHESIS_CHONDROITIN_SULFATE
14 - TYPE_I_DIABETES_MELLITUS
15 - ALPHA_LINOLENIC_ACID_METABOLISM
16 - RNA_DEGRADATION
17 - GRAFT_VERSUS_HOST_DISEASE
18 - BASAL_TRANSCRIPTION_FACTORS
19 - ETHER_LIPID_METABOLISM
20 - PRIMARY_BILE_ACID_BIOSYNTHESIS
21 - GLYOXYLATE_AND_DICARBOXYLATE_METABOLISM
22 - GLYCOSAMINOGLYCAN_BIOSYNTHESIS_KERATAN_SULFATE
23 - GLYCOSPHINGOLIPID_BIOSYNTHESIS_GANGLIO_SERIES
24 - SYSTEMIC_LUPUS_ERYTHEMATOSUS
25 - RIBOSOME
26 - SPLICEOSOME
27 - STEROID_HORMONE_BIOSYNTHESIS
28 - PATHOGENIC_ESCHERICHIA_COLI_INFECTION
29 - PORPHYRIN_AND_CHLOROPHYLL_METABOLISM
30 - AMINOACYL_TRNA_BIOSYNTHESIS
31 - PENTOSE_AND_GLUCURONATE_INTERCONVERSIONS
32 - VIBRIO_CHOLERAE_INFECTION
33 - CIRCADIAN_RHYTHM_MAMMAL
34 - OXIDATIVE_PHOSPHORYLATION
35 - GLYCOSYLPHOSPHATIDYLINOSITOL_GPI_ANCHOR_BIOSYNTHESIS
36 - MATURITY_ONSET_DIABETES_OF_THE_YOUNG
37 - DNA_REPLICATION
38 - N_GLYCAN_BIOSYNTHESIS
39 - PROTEIN_EXPORT
40 - COMPLEMENT_AND_COAGULATION_CASCADES
41 - PROXIMAL_TUBULE_BICARBONATE_RECLAMATION
42 - HEDGEHOG_SIGNALING_PATHWAY
43 - VALINE_LEUCINE_AND_ISOLEUCINE_DEGRADATION
44 - LYSINE_DEGRADATION
45 - NICOTINATE_AND_NICOTINAMIDE_METABOLISM
46 - FOLATE_BIOSYNTHESIS
47 - BIOSYNTHESIS_OF_UNSATURATED_FATTY_ACIDS
48 - PROTEASOME
49 - NUCLEOTIDE_EXCISION_REPAIR
50 - BASAL_CELL_CARCINOMA
51 - TYROSINE_METABOLISM
52 - STARCH_AND_SUCROSE_METABOLISM
53 - GLYCINE_SERINE_AND_THREONINE_METABOLISM
54 - METABOLISM_OF_XENOBIOTICS_BY_CYTOCHROME_P450
55 - ANTIGEN_PROCESSING_AND_PRESENTATION
56 - EPITHELIAL_CELL_SIGNALING_IN_HELICOBACTER_PYLORI_INFECTION
57 - LINOLEIC_ACID_METABOLISM
58 - SNARE_INTERACTIONS_IN_VESICULAR_TRANSPORT
59 - PARKINSONS_DISEASE
60 - HOMOLOGOUS_RECOMBINATION
61 - INTESTINAL_IMMUNE_NETWORK_FOR_IGA_PRODUCTION
62 - RETINOL_METABOLISM
63 - CARDIAC_MUSCLE_CONTRACTION
64 - PHENYLALANINE_METABOLISM
65 - DRUG_METABOLISM_CYTOCHROME_P450
66 - ABC_TRANSPORTERS
67 - FC_EPSILON_RI_SIGNALING_PATHWAY
68 - PRION_DISEASES
69 - LEISHMANIA_INFECTION
70 - GALACTOSE_METABOLISM
71 - CHEMOKINE_SIGNALING_PATHWAY
72 - LYSOSOME
73 - TGF_BETA_SIGNALING_PATHWAY

74 - RENIN_ANGIOTENSIN_SYSTEM
75 - TASTE_TRANSDUCTION
76 - ARACHIDONIC_ACID_METABOLISM
77 - GLYCEROLIPID_METABOLISM
78 - GAP_JUNCTION
79 - INSULIN_SIGNALING_PATHWAY
80 - STEROID_BIOSYNTHESIS
81 - ALANINE_ASPARTATE_AND_GLUTAMATE_METABOLISM
82 - PROPANOATE_METABOLISM
83 - PANTOTHENATE_AND_COA_BIOSYNTHESIS
84 - DORSO_VENTRAL_AXIS_FORMATION
85 - CYTOSOLIC_DNA_SENSING_PATHWAY
86 - NOTCH_SIGNALING_PATHWAY
87 - HUNTINGTONS_DISEASE
88 - TRYPTOPHAN_METABOLISM
89 - SPHINGOLIPID_METABOLISM
90 - PYRUVATE_METABOLISM
91 - PEROXISOME
92 - ENDOCYTOSIS
93 - INOSITOL_PHOSPHATE_METABOLISM
94 - NON_SMALL_CELL_LUNG_CANCER
95 - GLYCEROPHOSPHOLIPID_METABOLISM
96 - CITRATE_CYCLE_TCA_CYCLE
97 - TERPENOID_BACKBONE_BIOSYNTHESIS
98 - CYTOKINE_CYTOKINE_RECEPTOR_INTERACTION
99 - CELL_ADHESION_MOLECULES_CAMS
100 - TIGHT_JUNCTION
101 - RNA_POLYMERASE
102 - LEUKOCYTE_TRANSENDOTHELIAL_MIGRATION
103 - MELANOGENESIS
104 - FATTY_ACID_METABOLISM
105 - SMALL_CELL_LUNG_CANCER
106 - GNRH_SIGNALING_PATHWAY
107 - NEUROACTIVE_LIGAND_RECEPTOR_INTERACTION
108 - PENTOSE_PHOSPHATE_PATHWAY
109 - SELENOAMINO_ACID_METABOLISM
110 - SULFUR_METABOLISM
111 - NON_HOMOLOGOUS_END_JOINING
112 - ECM_RECEPTOR_INTERACTION
113 - REGULATION_OF_ACTIN_CYTOSKELETON
114 - PYRIMIDINE_METABOLISM
115 - VASCULAR_SMOOTH_MUSCLE_CONTRACTION
116 - DRUG_METABOLISM_OTHER_ENZYMES
117 - RENAL_CELL_CARCINOMA
118 - PHOSPHATIDYLINOSITOL_SIGNALING_SYSTEM
119 - CELL_CYCLE
120 - VIRAL_MYOCARDITIS
121 - GLYCOLYSIS_GLUCONEOGENESIS
122 - AXON_GUIDANCE
123 - CHRONIC_MYELOID_LEUKEMIA
124 - ALDOSTERONE_REGULATED_SODIUM_REABSORPTION
125 - ALZHEIMERS_DISEASE
126 - NATURAL_KILLER_CELL_MEDIATED_CYTOTOXICITY
127 - HYPERTROPHIC_CARDIOMYOPATHY_HCM
128 - PRIMARY_IMMUNODEFICIENCY
129 - JAK_STAT_SIGNALING_PATHWAY
130 - MTOR_SIGNALING_PATHWAY
131 - LONG_TERM_POTENTIATION
132 - B_CELL_RECEPTOR_SIGNALING_PATHWAY
133 - ARRHYTHMOGENIC_RIGHT_VENTRICULAR_CARDIOMYOPATHY_ARVC
134 - FOCAL_ADHESION
135 - FRUCTOSE_AND_MANNOSE_METABOLISM
136 - FC_GAMMA_R_MEDIATED_PHAGOCYTOSIS
137 - DILATED_CARDIOMYOPATHY
138 - GLUTATHIONE_METABOLISM
139 - PURINE_METABOLISM
140 - VALINE_LEUCINE_AND_ISOLEUCINE_BIOSYNTHESIS
141 - BASE_EXCISION_REPAIR
142 - VASOPRESSIN_REGULATED_WATER_REABSORPTION
143 - GLIOMA
144 - LONG_TERM_DEPRESSION
145 - TOLL_LIKE_RECEPTOR_SIGNALING_PATHWAY
146 - UBIQUITIN_MEDIATED_PROTEOLYSIS
147 - T_CELL_RECEPTOR_SIGNALING_PATHWAY
148 - OTHER_GLYCAN_DEGRADATION
149 - MAPK_SIGNALING_PATHWAY

150 - ARGININE_AND_PROLINE_METABOLISM
151 - OOCYTE_MEIOSIS
152 - GLYCOSPHINGOLIPID_BIOSYNTHESIS_LACTO_AND_NEOLACTO_SERIES
153 - PATHWAYS_IN_CANCER
154 - ADIPOCYTOKINE_SIGNALING_PATHWAY
155 - WNT_SIGNALING_PATHWAY
156 - MELANOMA
157 - NEUROTROPHIN_SIGNALING_PATHWAY
158 - TAURINE_AND_HYPOTAURINE_METABOLISM
159 - VEGF_SIGNALING_PATHWAY
160 - AMINO_SUGAR_AND_NUCLEOTIDE_SUGAR_METABOLISM
161 - CYSTEINE_AND_METHIONINE_METABOLISM
162 - RIG_I_LIKE_RECEPTOR_SIGNALING_PATHWAY
163 - AMYOTROPHIC_LATERAL_SCLEROSIS_ALS
164 - ENDOMETRIAL_CANCER
165 - PROSTATE_CANCER
166 - GLYCOSPHINGOLIPID_BIOSYNTHESIS_GLOBO_SERIES
167 - PANCREATIC_CANCER
168 - NITROGEN_METABOLISM
169 - PPAR_SIGNALING_PATHWAY
170 - MISMATCH_REPAIR
171 - ADHERENS_JUNCTION
172 - HEMATOPOIETIC_CELL_LINEAGE
173 - PROGESTERONE_MEDIATED_OOCYTE_MATURATION
174 - COLORECTAL_CANCER
175 - CALCIUM_SIGNALING_PATHWAY
176 - ERBB_SIGNALING_PATHWAY
177 - ONE_CARBON_POOL_BY_FOLATE
178 - GLYCOSAMINOGLYCAN_DEGRADATION
179 - BLADDER_CANCER
180 - TYPE_II_DIABETES_MELLITUS
181 - NOD_LIKE_RECEPTOR_SIGNALING_PATHWAY
182 - APOPTOSIS
183 - RIBOFLAVIN_METABOLISM
184 - ACUTE_MYELOID_LEUKEMIA
185 - P53_SIGNALING_PATHWAY
186 - THYROID_CANCER

Table S1 The distribution metrics for the pathway-averaged L^{tr} and N^{tr} values among the cancer and other pathways, corresponding to the boxplots in Figure 1A,B

measures	pathway-averaged L^{tr} boxplots (Fig. 1A)		pathway-averaged N^{tr} boxplots (Fig. 1B)	
	other	cancer	other	cancer
minimum	6250	50610	1.142	2.073
1st quartile	37140	75730	1.827	2.371
median	52630	82750	2.069	2.535
mean	61420	86250	2.079	2.597
3rd quartile	80480	95260	2.307	2.791
maximum	203600	136100	3.413	3.345

Table S2 The KEGG pathway enrichment analysis for the genes significantly associated with autism spectrum disorder (ASD)

Genes significantly associated with ASD [FDR<0.05]		
**KEGG pathway**	**Number of genes**	**p^{EASE} score**
Cell adhesion molecules, CAMs	5	$1.84\ 10^{-3}$
Neuroactive ligand-receptor interaction	6	$3.25\ 10^{-3}$
Long-term potentiation	3	$3.14\ 10^{-2}$
Long-term depression	3	$3.23\ 10^{-2}$
Calcium signaling pathway	4	$3.43\ 10^{-2}$

The significantly enriched KEGG pathways are revealed via DAVID gene functional annotation server, taking *Homo sapiens* as a correction background. The p^{EASE} significance scores for the enrichment are shown along with the number of hit genes. The full list of genes that appear in each enriched pathway can be found in Additional file 3. The gene set is taken from *King et al, Nature, 501:58-62, 2013*.

Table S3 The KEGG pathway enrichment analysis for the top genes by summed exon length

Genes with longest total exon		
**KEGG pathway**	**Number of genes**	**p^{EASE} score**
Focal adhesion	30	$6.69\ 10^{-8}$
ECM-receptor interaction	18	$3.28\ 10^{-7}$
Calcium signaling pathway (*,#)	25	$2.77\ 10^{-6}$
Long-term potentiation (*)	12	$3.46\ 10^{-4}$
Long-term depression (*)	12	$3.94\ 10^{-4}$
Pathways in cancer (+)	31	$4.26\ 10^{-4}$
MAPK signaling pathway (+)	26	$9.37\ 10^{-4}$
Hypertrophic cardiomyopathy, HCM (#)	12	$2.33\ 10^{-3}$
Arrhythmogenic right ventricular cardiomyopathy (#)	11	$3.23\ 10^{-3}$
Dilated cardiomyopathy (#)	12	$4.37\ 10^{-3}$
Small cell lung cancer (+)	11	$6.67\ 10^{-3}$
Type II diabetes mellitus (**)	8	$6.67\ 10^{-3}$
Vascular smooth muscle contraction	13	$7.23\ 10^{-3}$
Gap junction	11	$9.95\ 10^{-3}$
Wnt signaling pathway	15	$1.34\ 10^{-2}$
Axon guidance (*)	13	$2.08\ 10^{-2}$
Phosphatidylinositol signaling system	9	$2.50\ 10^{-2}$
Fatty acid biosynthesis	3	$3.05\ 10^{-2}$
Colorectal cancer (+)	9	$4.81\ 10^{-2}$

The genes with summed exon length greater than the all-data median by twice the standard deviation are used. The significantly enriched KEGG pathways are revealed via DAVID gene functional annotation server, taking *Homo sapiens* as a correction background. The p^{EASE} significance scores for the enrichment are shown along with the number of hit genes. The notations in the brackets mark the pathways linked to cancer (+), neurological (*), cardiological (#) and other (**) multigenic pathological conditions. The full list of genes that appear in each enriched pathway can be found in Additional file 3.

Table S4 The KEGG pathway enrichment analysis for the top genes by transcript length

**Genes with longest transcript**		
**KEGG pathway**	**Number of genes**	**p^{EASE} score**
Calcium signaling pathway (*,#)	27	$5.61 \cdot 10^{-9}$
Axon guidance (*)	23	$6.09 \cdot 10^{-9}$
Long-term potentiation (*)	16	$5.18 \cdot 10^{-8}$
Long-term depression (*)	16	$6.39 \cdot 10^{-8}$
Vascular smooth muscle contraction	16	$3.76 \cdot 10^{-5}$
Arrhythmogenic right ventricular cardiomyopathy (#)	13	$4.46 \cdot 10^{-5}$
Hypertrophic cardiomyopathy, HCM (#)	13	$1.37 \cdot 10^{-4}$
Phosphatidylinositol signaling system	12	$1.63 \cdot 10^{-4}$
Gap junction	13	$2.15 \cdot 10^{-4}$
Dilated cardiomyopathy (#)	13	$2.95 \cdot 10^{-4}$
Neuroactive ligand-receptor interaction (*)	23	$5.90 \cdot 10^{-4}$
ErbB signaling pathway	12	$6.90 \cdot 10^{-4}$
GnRH signaling pathway	12	$1.87 \cdot 10^{-3}$
Cell adhesion molecules, CAMs	14	$2.46 \cdot 10^{-3}$
Tight junction	14	$2.82 \cdot 10^{-3}$
Purine metabolism	15	$3.38 \cdot 10^{-3}$
MAPK signaling pathway (+)	21	$5.34 \cdot 10^{-3}$
Focal adhesion	17	$7.03 \cdot 10^{-3}$
Chondroitin sulfate biosynthesis	5	$1.08 \cdot 10^{-2}$
Regulation of actin cytoskeleton	17	$1.30 \cdot 10^{-2}$
Fc gamma R-mediated phagocytosis	10	$1.42 \cdot 10^{-2}$
Heparan sulfate biosynthesis	5	$1.94 \cdot 10^{-2}$
Alzheimer's disease (**)	13	$3.19 \cdot 10^{-2}$
Type II diabetes mellitus (**)	6	$4.01 \cdot 10^{-2}$

The genes with longest transcript length greater than the all-data median by twice the standard deviation are used. The significantly enriched KEGG pathways are revealed via DAVID gene functional annotation server, taking *Homo sapiens* as a correction background. The p^{EASE} significance scores for the enrichment are shown along with the number of hit genes. The notations in the brackets mark the pathways linked to cancer (+), neurological (*), cardiological (#) and other (**) multigenic pathological conditions. The full list of genes that appear in each enriched pathway can be found in Additional file 3.

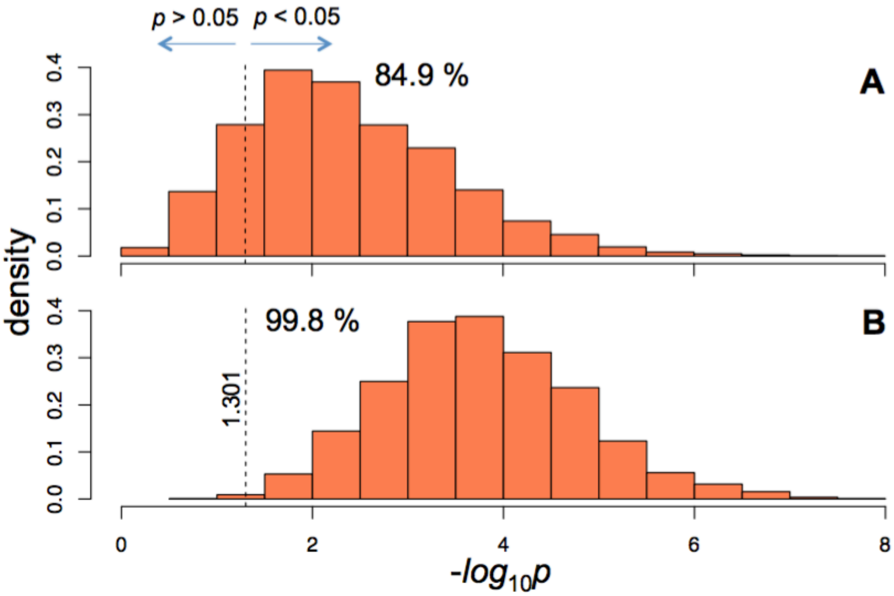

Figure S1 Further assessment of the shift significance while comparing pathway-averaged L^{tr} and N^{tr} values in cancer versus other pathways. The histograms present the distributions of the *p*-values while comparing the pathway-averaged L^{tr} **(A)** and N^{tr} **(B)** of the 15 cancer pathways with 15 (equal number) other pathways randomly selected from the available 171. The random selection was done 100000 times, resulting in the above *p*-value distributions brought in $-log_{10}$ scale, where, for instance, the value 4 means $p=10^{-4}$. The dotted vertical lines outline $p=0.05$ ($-log_{10}p=1.301$), used as a significance threshold. As can be inferred from the figure, such analysis resulted in significant *p*-values for L^{tr} and N^{tr} 84.9% and 99.8% of times respectively. Please note, that this test is done as a direct demonstration of the absence of the size difference bias in the significance of the shifts between the cancer vs. other distributions. However, the size difference is reflected in the used *p*-value analyses without the enforced size equalisation, due to the negative (*p*-value increase) effect of the low data numbers in either of the distributions, reducing the confidence on the corresponding mean value.

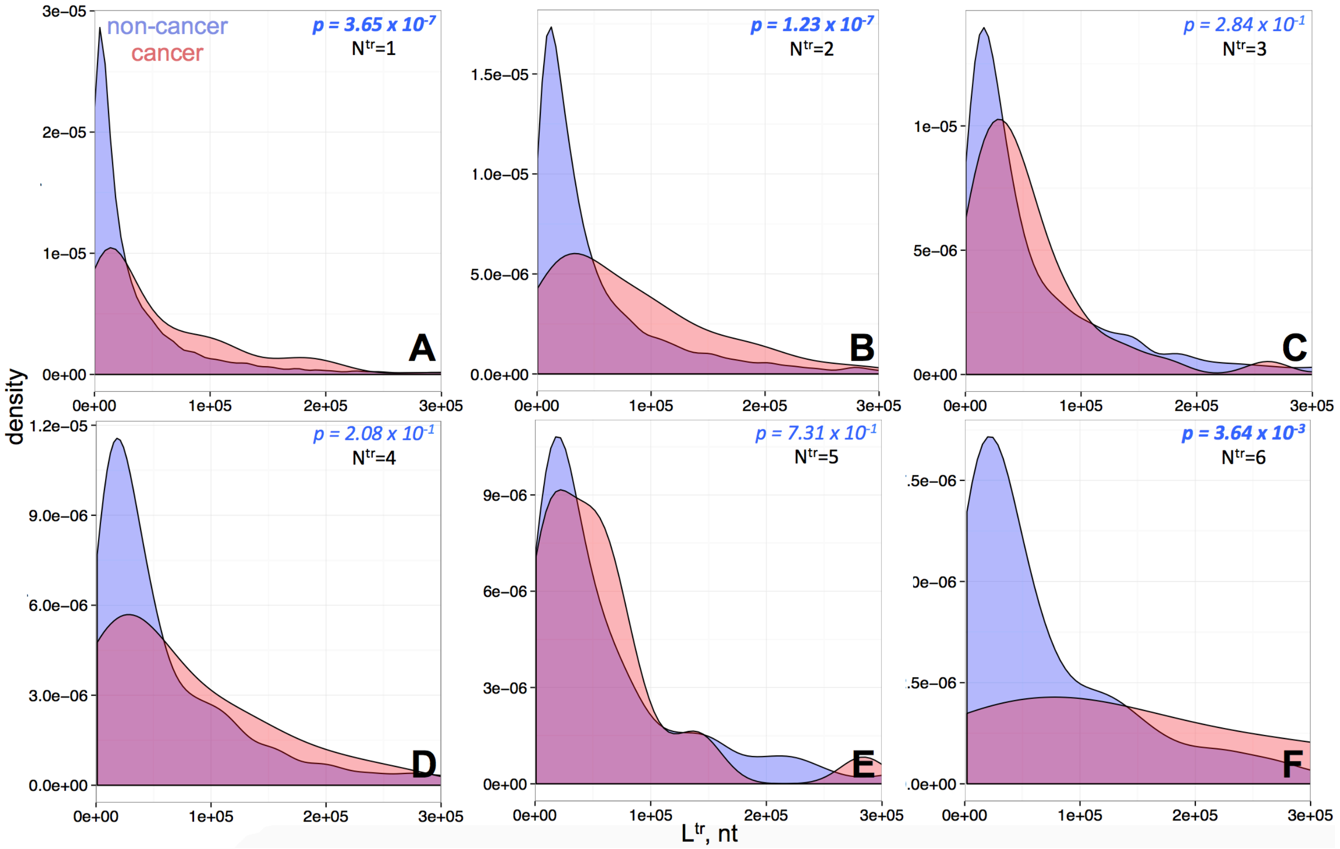

Figure S2 Distributions of the longest transcript length L^{tr} in cancer (red) and other (blue) pathways for the genes with different N^{tr} number of transcripts. The plots **A-F** are for the N^{tr} of 1 to 6. The *p*-values reflecting on the significance of a positive shift in the distributions for the cancer pathways are shown on top of each plot. The number of genes in both distribution are {170, 10795}, {91, 3841}, {41, 1964}, {27, 991}, {24, 515} and {9, 313} for the plots **A-F** correspondingly, brought in the {cancer/red, other/blue} format.

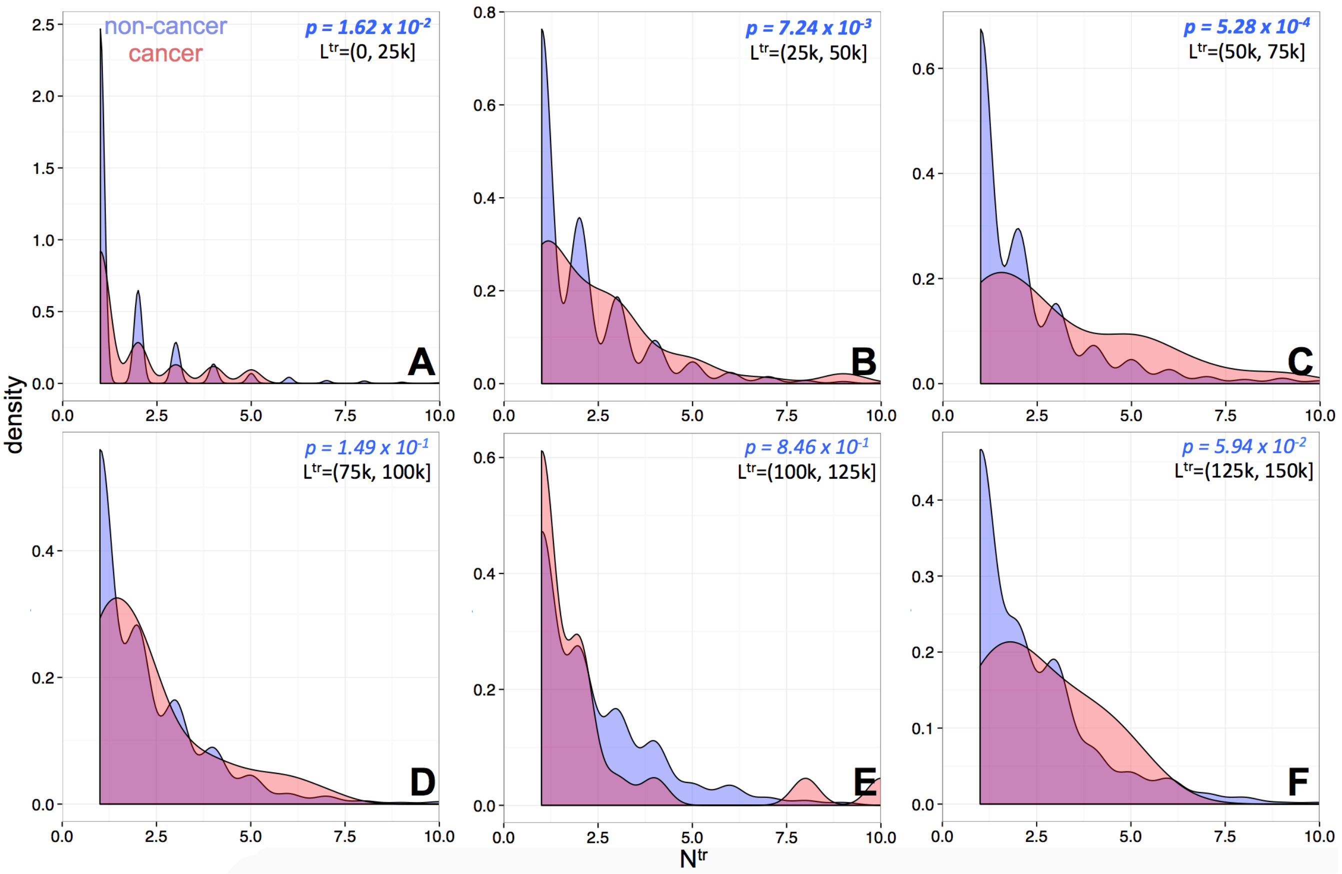

Figure S3 Distributions of the N^{tr} number of transcripts in cancer (red) and other (blue) pathways for the genes with different intervals of L^{tr} longest transcript length. The *p*-values reflecting on the significance of a positive shift in the distributions for the cancer pathways, along with the L^{tr} interval are shown on top of each plot. The density values (y-axes) peak at discrete integer N^{tr} (x-axes), with the intermediate values filled due to the smoothening at the density calculation procedure. The latter has no effect on the *p*-values that were estimated based on the actual N^{tr} values, independent from the density calculations. The number of genes in both distribution are {133, 9920}, {64, 3383}, {43, 1662}, {40, 954}, {24, 687} and {18, 478} for the plots **A-F** correspondingly, brought in the {cancer/red, other/blue} format.

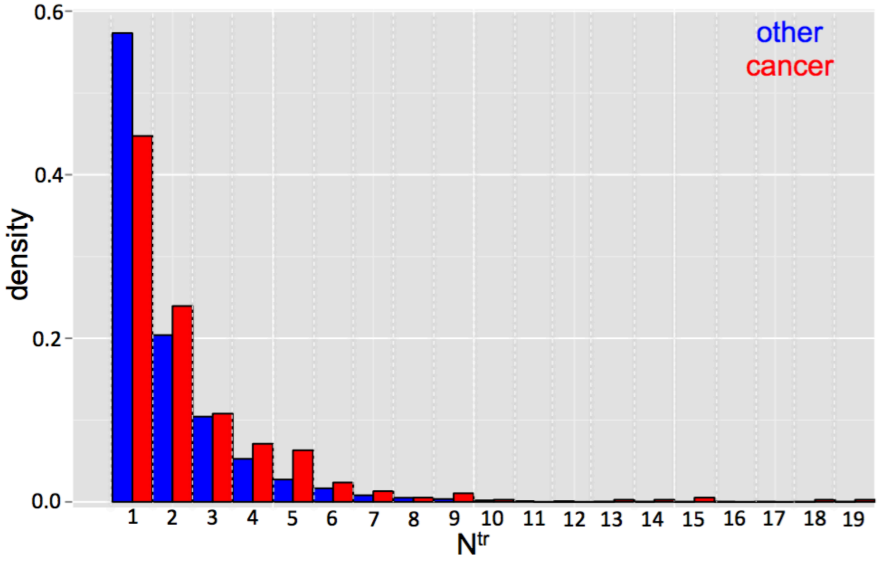

Figure S4 Distributions of the N^{tr} number of transcripts in cancer (red) and other (blue) pathways for all the genes. The plot is the combined and discrete version of Figure S3A-F.

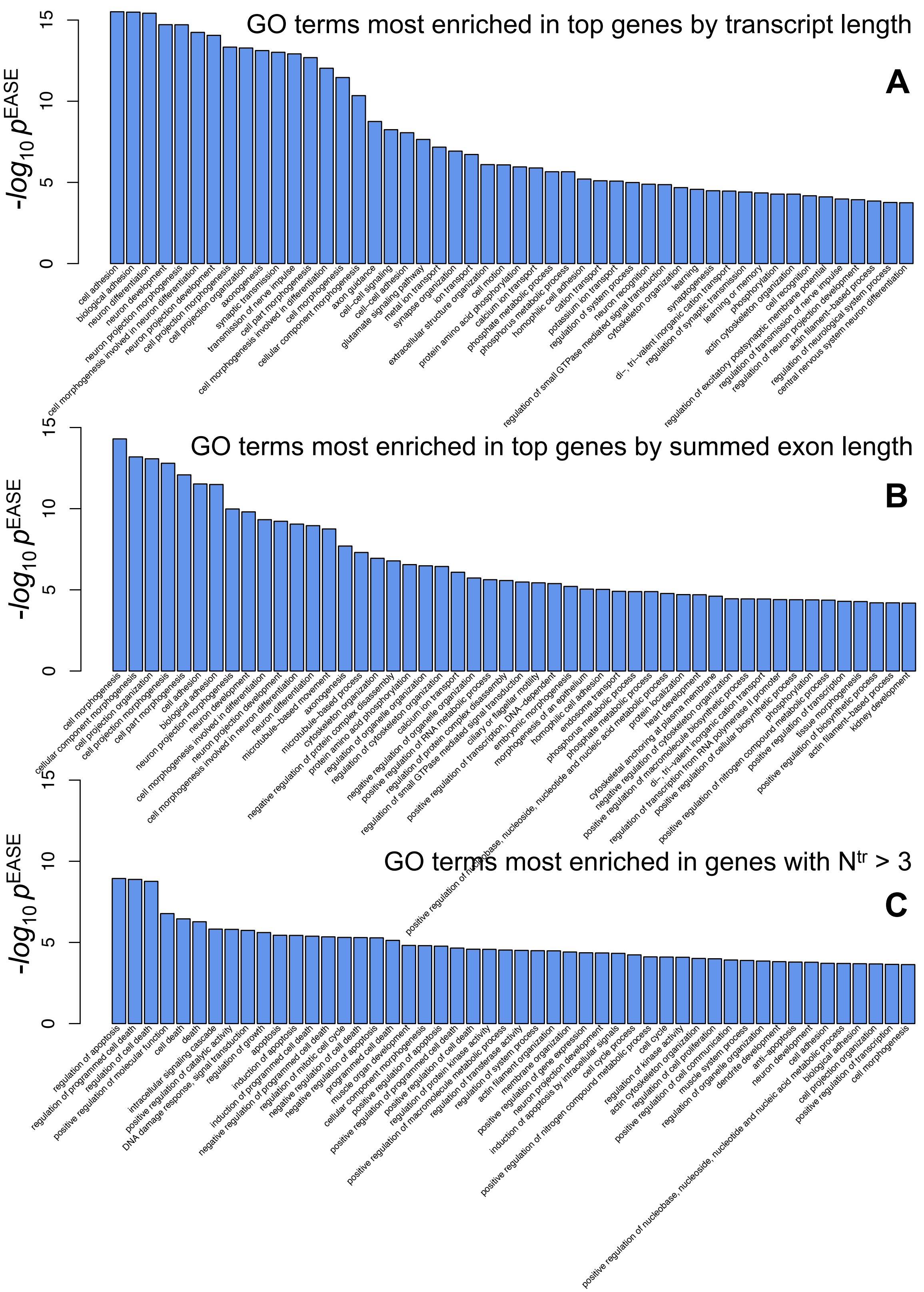

Figure S5 Gene ontology (GO) analyses for the genes special in length and splicing complexity. The 50 most enriched GO terms (BP set) are shown for the top genes by longest transcript length **(A)**, top genes by summed exon length **(B)**, and genes with greater than 3 transcript variants **(C)**. The y-axis shows the p^{EASE} score for the enrichment significance in a $-log_{10}$ scale ($-log_{10}p^{EASE}>1.301$ means $p^{EASE}<0.05$). The full set of significant GO terms and gene lists is presented in Additional file 3.

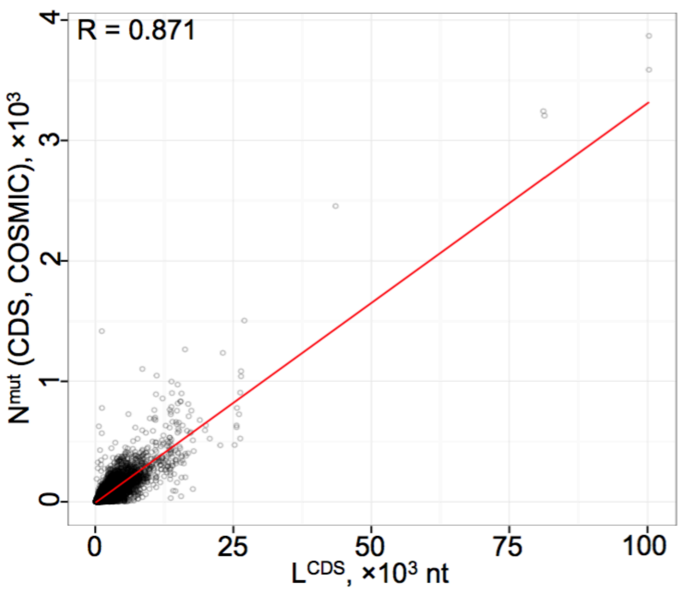

Figure S6 Correlation between the overall number of cancer-linked mutations in the coding sequences (CDS) of different genes and their CDS length. The cancer-linked mutations (as deposited in the COSMIC database) are counted from only the coding regions. The correlation coefficient (top-left corner) and the linear mode fit (red line) are shown.

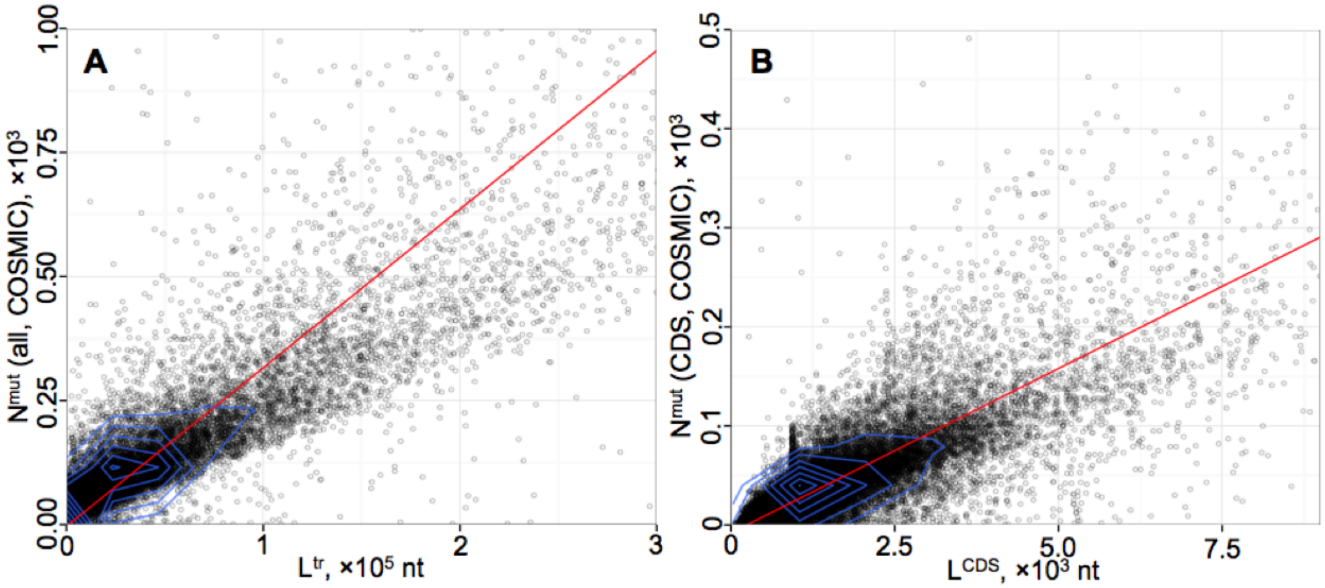

Figure S7 Plots representing the zoomed versions of Figure 5A (A) and Figure S6 (B). Both **A** and **B** graphs detail the lower-left corner of the corresponding original plots, with additional contour lines (blue) added to illustrate the distribution of data points in the crowded region.
